# Comparison of HCV Prevalence in Pakistan and Iran; An Insight into Future

**DOI:** 10.5812/hepatmon.11466

**Published:** 2014-01-12

**Authors:** Muhammad Sohail Afzal, Tahir Ahmed, Najam us Sahar Sadaf Zaidi

**Affiliations:** 1 Atta ur Rahman School of Applied Biosciences, National University of Science and Technology, Islamabad, Pakistan

**Keywords:** Hepatitis C, Infection, Prevalence, Awareness, Health Promotion

*Dear Editor,*

A recent report by Shakeri et al. (March, 2013) published in Hepatitis Monthly showed that HCV prevalence is 0.2% in Mashad, Iran (1). Moreover, Previous report from Iran showed the similar prevalence of HCV in voluntaries blood donors (2). Pakistan, a neighboring country of Iran, is a low socioeconomic country having more than 10 million HCV infected people (3). Recently we have published the prevalence of HCV among 62,251 healthy blood donors from North West Province of Pakistan. The study showed that 2.6 % of healthy individuals were HCV antibody positive (4). We also concluded that the HCV prevalence is increasing with time when the results were compared with other previous reports. 

HCV with more than 180 million infections worldwide (5), can lead to chronic infection and hepatocellular carcinoma (HCC) in 60% to 80% of infected individuals (6). Genetic differences among different ethnic groups results in differential susceptibility of infection and 20 % of infected individuals can clear the virus naturally due to their genetic makeup (7, 8). Better understanding of genetic factors, which are differentially distributed among different ethnic groups and have effect on hepatitis C susceptibility, progression, pathogeneses, would provide a scientific basis for HCV differential prevalence. Complete understanding the underlying molecular mechanisms involved in immune regulation in HCV infected patients will help in the development of new immunomodulatory treatments. 

Currently there is no vaccine available to control HCV largely due to its unpredictable genomic variations. As Pakistani population is more affected with this deadly virus, a large proportion of infected individuals are in danger of developing cirrhosis and hepatocellular carcinoma. In Iran, the situation in not alarming currently but there is a need to conduct the continuous and regular prevalence studies on larger scale. As the incidence of HCV infection in Pakistan is increasing with time, the HCV prevalence studies are recommended in Iran on regular intervals to estimate the actual infection rate of HCV. Massive awareness campaigns are also needed to educate the general population about the roots of HCV spread and prevention methods to contain the deadly virus spread. It is observed that barbers and dentists are the major cause of viral spread in healthy population. Therefore, it is necessary to educate the barbers and dentists in Iran and their practices should be supervised more efficiently according to HCV safety protocols.
